# Papain-Catalyzed Synthesis of Polyglutamate Containing a Nylon Monomer Unit

**DOI:** 10.3390/polym8050194

**Published:** 2016-05-13

**Authors:** Kenjiro Yazawa, Keiji Numata

**Affiliations:** Enzyme Research Team, RIKEN Center for Sustainable Resource Science, 2-1 Hirosawa, Wakoshi, Saitama 351-0198, Japan; kenjiro.yazawa@riken.jp

**Keywords:** chemoenzymatic synthesis, aminolysis, peptide, papain, bromelain, proteinase K, lipase, thermal property, nylon

## Abstract

Peptides have the potential to serve as an alternative for petroleum-based polymers to support a sustainable society. However, they lack thermoplasticity, owing to their strong intermolecular interactions. In contrast, nylon is famous for its thermoplasticity and chemical resistance. Here, we synthesized peptides containing a nylon unit to modify their thermal properties by using papain-catalyzed chemoenzymatic polymerization. We used l-glutamic acid alkyl ester as the amino acid monomer and nylon 1, 3, 4, and 6 alkyl esters as the nylon unit. Papain catalyzed the copolymerization of glutamic acid with nylon 3, 4, and 6 alkyl esters, whereas the nylon 1 unit could not be copolymerized. Other proteases used in this study, namely, bromelain, proteinase K, and *Candida antarctica* lipase (CALB), were not able to copolymerize with any nylon units. The broad substrate specificity of papain enabled the copolymerization of l-glutamic acid with a nylon unit. The peptides with nylon units demonstrated different thermal profiles from that of oligo(l-glutamic acid). Therefore, the resultant peptides with various nylon units are expected to form fewer intermolecular hydrogen bonds, thus altering their thermal properties. This finding is expected to broaden the applications of peptide materials and chemoenzymatic polymerization.

## 1. Introduction

Peptides have received attention as eco-friendly alternatives for petroleum-based materials because of their unique physical properties and biological functions. For human use of peptides as bulk materials , it would be expected that peptide-based materials would need to be fabricated via a thermal process without any organic solvents [1]. However, peptides do not have a melting point but instead show decomposition during the heating process because the intermolecular hydrogen bonds in peptides are so strong that the intrachain covalent bonds begin to degrade before the melting transition [2]. To design thermally processable peptide materials, we therefore focus on the copolymerization of peptides and unnatural components. In this study, we selected nylon units as a peptide partner because nylon has an amide bond and has characteristics of thermoplasticity and chemical resistance. In addition, nylon 4 is an attractive unit because of its biomass-based origin and biodegradability in natural environment [3]. Thus, the introduction of nylon units into peptides changes the density of hydrogen bonds between the peptide main chains and can lead to new functions such as peptides with unique thermal properties.

To synthesize peptides, solid phase peptide synthesis requiring protection-deprotection procedures is generally used [4,5,6]. Peptides can also be biosynthesized by using recombinant DNA expression systems, in which amino acid sequences influence protein yield as well as productivity, and multistep purifications are necessary [7,8,9,10]. In this study, we focused on chemoenzymatic peptide synthesis to copolymerize amino acid and nylon units. Chemoenzymatic peptide synthesis is a hydrolase-catalyzed polymerization that has the possibility of introducing unnatural amino acids and artificial units [11,12,13,14], and it is a clean, mild, stereoselective reaction with a high atom economy [14]. Proteases generally function as hydrolases, and they are also able to catalyze aminolysis. The aminolysis can proceed kinetically when excess substrate amino acids are present around the enzyme. Papain, a cysteine protease, shows broad substrate specificity and exhibits endopeptidase, amidase, and esterase activities, which have been widely used in chemoenzymatic synthesis [15,16,17,18,19,20,21]. Beer *et al.* have provided the first demonstration that papain can recognize the nylon 3 monomer β-alanine as a substrate in a dipeptide synthesis [16]. Thus, papain is expected to incorporate unnatural amino acids such as nylon monomers. Here, we used papain to synthesize peptides with nylon units. We also used other enzymes, namely bromelain, proteinase K, and *Candida antarctica* lipase B (CALB). Bromelain is a cysteine protease with broad substrate specificity and is frequently used in chemoenzymatic synthesis [22,23,24,25,26]. Proteinase K is a serine protease that has affinity not only for the peptide bond next to the carboxyl group of aliphatic amino acids but also for esters [27,28,29]. CALB is a lipolytic enzyme that catalyzes many reactions, such as esterification, amidation, and transesterification [30,31,32,33,34,35]. Lipase-catalyzed amide bond formations have been reported by Loos *et al.* [35] and Cheng *et al.* [36]. We compared the reaction efficiencies of the four enzymes used in this study.

**Figure 1:**
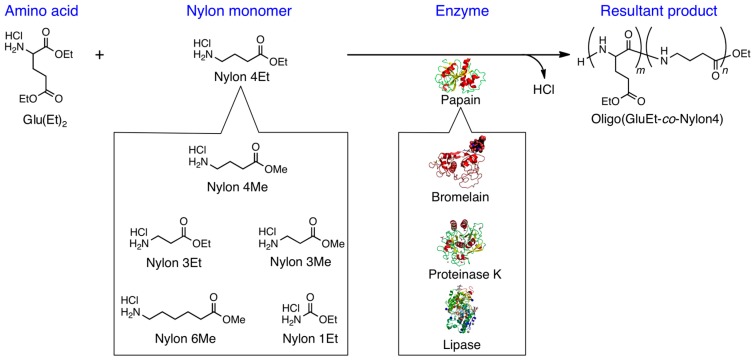
Schematic illustration of the copolymerization of amino acids and nylon monomers by chemoenzymatic synthesis. Glu(Et)_2_ was used as a model amino acid, whereas six types of nylon monomer alkyl esters with various chain lengths were used as the nylon unit. Chemoenzymatic synthesis was performed using four enzymes, and their reaction efficiencies were investigated.

As a model amino acid monomer, we used l-glutamic acid diethyl ester (Glu(Et)_2_) because of its relatively high reactivity. We selected four types of commercially available nylon monomers—nylon 1 [ethyl carbamate (nylon 1Et)], nylon 3 [β-alanine methyl ester hydrochloride (nylon 3Me), β-alanine ethyl ester hydrochloride (nylon 3Et)], nylon 4 [methyl 4-aminobutyrate hydrochloride (nylon 4Me), ethyl 4-aminobutyrate hydrochloride (nylon 4Et)], and nylon 6 [methyl 6-aminohexanoate hydrochloride (nylon 6Me)]—to copolymerize with Glu(Et)_2_ (Figure 1). In particular, nylon 4 is biomass-based and biodegradable, and it is considered to be an eco-friendly bioplastic [3]. However, the process window (gap between melting and degradation temperatures) is too small to allow practical industrial processing. Copolymers of the nylon 4 unit and amino acids can be used as an eco-friendly and biomass-based material.

In this study, oligo glutamic acid-γ-ethyl ester (oligo(GluEt)) with a nylon unit, which was synthesized by chemoenzymatic copolymerization, was characterized by ^1^H nuclear magnetic resonance (NMR) and matrix assisted laser desorption ionization time of flight mass spectrometry (MALDI-TOF MS) to confirm the resultant compositions. The thermal properties of oligo(GluEt) with a nylon unit were also characterized by thermogravimetric analysis (TGA) and differential scanning calorimetry (DSC) to evaluate the effect of the nylon unit on the thermal properties of the peptides. From these results, the relationship between the nylon units and thermal properties was determined, considering the density of hydrogen bonds between the peptide main chains.

## 2. Materials and Methods

### 2.1. Materials

l-Glutamic acid diethyl ester hydrochloride, methyl 4-aminobutyrate hydrochloride (nylon 4Me), ethyl 4-aminobutyrate hydrochloride (nylon 4Et), β-alanine methyl ester hydrochloride (nylon 3Me), β-alanine ethyl ester hydrochloride (nylon 3Et), methyl 6-aminohexanoate hydrochloride (nylon 6Me), 2-(4-hydroxyphenylazo) benzoic acid (HABA), sodium trifluoroacetate (Na-TFA), and *Candida antarctica* lipase B (CALB) were purchased from Sigma-Aldrich (St. Louis, MO, USA). Ethyl carbamate (nylon 1Et), papain, bromelain, and proteinase K were purchased from Wako Pure Chemical Industries (Osaka, Japan). All of the chemicals and enzymes were used without any purification. The activity of papain was 0.0015 U/mg, and one unit was defined to hydrolyze *N*α-benzoyl-l-arginine-*p*-nitroanilide hydrochloride and release 1 µmol of *p*-nitroaniline per minute at a pH of 7.5 and 25 °C. The activity of bromelain was 1090 U/mg, and one unit was defined to hydrolyze *N*α-carbobenzoxy-l-lysine-*p*-nitrophenyl ester and release 1 µmol of *p*-nitrophenol per minute at a pH of 4.6 and 25 °C. The activity of proteinase K was 23 U/mg, and one unit was defined to hydrolyze urea-denatured hemoglobin and to produce color equivalent to 1 µmol of tyrosine per minute at a pH of 7.5 and 37 °C. The activity of lipase was 819 U/mg, and one unit was defined to hydrolyze triacylglycerol and release 1 µmol of fatty acid per hour at a pH of 7.2 and 37 °C.

### 2.2. Chemoenzymatic Peptide Synthesis

The reaction conditions were slightly modified from those in a previous report on the chemoenzymatic synthesis of oligo(GluEt) catalyzed by papain [17]. Briefly, a molar excess of nylon monomer (0.54 M) and Glu(Et)_2_ (0.06 M) were mixed with 20 mg/mL papain, bromelain, proteinase K, or lipase in 5 mL of 1 M phosphate buffer at a pH of 8.0. The chemoenzymatic reactions were performed at 40 °C for 12 h and stirred at 1000 rpm using an EYELA ChemiStation (Tokyo, Japan). The resultant white precipitate was centrifuged at 12,000× *g* for 5 min and was washed with diluted HCl (pH = 2) twice and with Milli-Q water. The products were lyophilized.

### 2.3. MALDI-TOF MS

The MALDI-TOF MS spectra were obtained using an Autoflex Speed MALDI-TOF-MS system spectrophotometer (Bruker, Bremen, Germany) operating in linear positive mode. The lyophilized product (0.4 mg/mL) was dissolved in TA solution, consisting of 0.1% trifluoroacetic acid, 80% acetonitrile, and 20% water. Then, the sample solution was mixed with 20 mg/mL HABA and 1 mg/mL Na-TFA, which were used as the matrix and cationic reagent, respectively. The prepared sample (1 µL) was spotted on the target plate and dried at an ambient temperature. The acquired data were analyzed with FLEX analysis software (Bruker, Bremen, Germany).

### 2.4. ^1^H-NMR

^1^H-NMR was recorded on a Varian NMR System 500 (500 MHz) spectrometer (Varian Medical Systems, Palo Alto, CA, USA) at 25 °C controlled with VnmrJ software (Agilent Technologies, Santa Clara, CA, USA). The lyophilized samples were dissolved in dimethyl sulfoxide-*d*_6_ (DMSO-*d*_6_) at a concentration of 10 mg/mL. One hundred and twenty-eight scans were recorded during each ^1^H-NMR measurement. Data were processed and analyzed using ACD/NMR Processor Academic Edition, version 12.01 (Advanced Chemistry Development, Toronto, ON, Canada).

### 2.5. Thermal Analysis

TGA and DSC measurements were performed simultaneously by using a TGA/DSC2 (Mettler Toledo, Greifensee, Switzerland) according to a previous study [37]. The sample synthesized by chemoenzymatic polymerization (5–7 mg) was weighed and sealed in an aluminum pan. The lid of the aluminum pan had a pinhole to prevent the pan from bursting because of an increase in the internal pressure during the heating process. The product was heated at 10 °C/min from 30 to 400 °C under a nitrogen atmosphere in triplicate. The device was calibrated with an empty cell to form a baseline and with indium to characterize the heat flow and temperature of the system.

## 3. Results and Discussion

### 3.1. Papain-Catalyzed Copolymerization of Peptides with a Nylon 4 Unit

**Figure 2:**
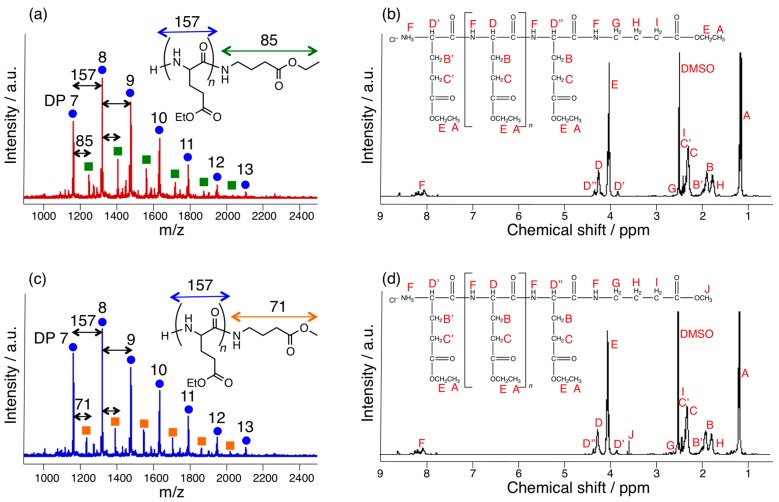
MALDI-TOF MS (**a**,**c**) and ^1^H–NMR (**b**,**d**) spectra of the papain-catalyzed reaction product using Glu(Et)_2_ with nylon 4Et (**a**,**b**) or nylon 4Me (**c**,**d**) as substrates. Mass spectra were obtained in the linear positive mode using HABA and Na-TFA as the matrix and cationic reagent, respectively. Peaks with blue circles denote oligo(GluEt) with various DP, whereas peaks with green (**a**) or orange (**c**) squares denote oligo(GluEt) with a nylon 4Et or nylon 4Me, respectively. DMSO-*d*_6_ was used as the solvent in NMR measurements.

Methyl and ethyl esters of monomeric substrates were used in this study because of their relatively higher reactivities in comparison to those of the free amino acids and nylon units [15,28]. Because the *pK*_a_ of substrate monomer is the main determinant of the reaction efficiency in the chemoenzymatic polymerizatrion, yield and degree of polymerization are largely dependent on the pH of the reaction medium [14]. Therefore, we set the pH of 8 using phosphate buffer, which is within the range of optimal condition for the chemoenzymatic synthesis by papain [17], bromelain [38], proteinase K [27], and lipase [39]. Furthermore, we set at 40 °C for the aminolysis reaction, because the aminolysis activity was larger at 40 °C in the polymerization by papain [17], bromelain [38], and proteinase K [27]. In addition, we found that the substrate monomer, namely l-glutamic acid diethyl ester, tends to be hydrolyzed when the reaction temperature was set over 40 °C [29]. Therefore, we used pH 8 and 40 °C for the reaction conditions. As the first step, we performed papain-catalyzed chemoenzymatic synthesis using Glu(Et)_2_ and nylon 4Et and then obtained a resultant white precipitate at a yield of 53% based on the initial amounts of the monomers used in the reaction. The MALDI-TOF MS spectrum of the resultant product is shown in Figure 2a. Peaks with constant intervals of 157 g/mol were detected, thus indicating that oligo(GluEt) was successfully synthesized with DP from 7 to 12. In this study, the limiting factor of polymer length is mainly determined by the solubility of the resultant product in the reaction medium. When peptide chains elongate to a length at which they are insoluble, they precipitate from solution, which prevents the increase of the molecular weight. In addition, the other peaks with an interval of 85 g/mol apart from the peaks derived from oligo(GluEt) indicated that oligo(GluEt) and only one nylon 4 unit were copolymerized. We did not detect any peaks derived from peptides containing more than two nylon 4 units. The resultant product was also characterized by ^1^H-NMR (Figure 2b), and nylon-derived peaks were detected in addition to the peaks assigned to oligo(GluEt). From the ^1^H-NMR results, approximately 1 out of 10 oligo(GluEt) had a nylon 4Et unit, according to the integral values of the peaks corresponding to the N-terminal α-proton and C-terminal methylene proton shown as D’ and G, respectively, in Figure 2b. The formation of γ-glutamate linkage in the oligomer was not detected, based on the ^1^H–NMR of the resultant product and a previous report on poly(α-methyl γ-l-glutamate) [40]. Furthermore, the oligomers formed from diethyl l-glutamate hydrochloride using papain were exclusively α-linked, which was confirmed by ^1^H-^1^H COSY NMR [41]. In addition, the papain-catalyzed polymerization of γ-methyl l-glutamate resulted in no occurrence of the polymerization [41]. These data indicate that the poly-α-glutamate was exclusively produced in the papain-catalyzed synthesis. The MALDI-TOF MS spectra showed a negligible amount of free pendant carboxylic acid in the resultant product. The major series of signals were derived from the product with ester groups. According to the ^1^H–NMR spectra, the ratio of the integration value between the peaks derived from the N-terminal α-proton and the C-terminal ester group was nearly identical, which indicates that almost all the ester groups maintained. Thus, our resultant product was mainly composed of the ester moiety at the C-terminus. This is probably because the resultant product was formed as precipitate, which limits accessibility of papain to the resultant product and prevents the hydrolysis of the ester bonds.

**Scheme 1:**
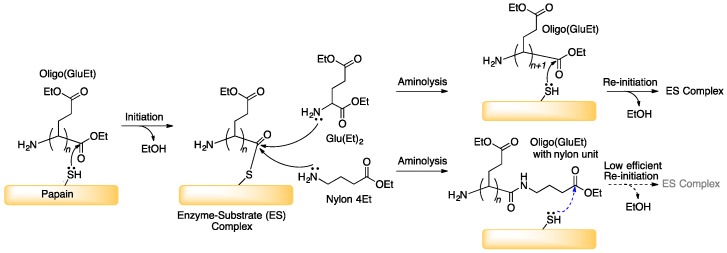
Reaction scheme of the papain-catalyzed synthesis of peptides with a nylon unit.

To clarify the difference between nylon ethyl ester and nylon methyl ester, we additionally used nylon 4Me as a nylon unit, which resulted in a reaction yield of 45%. The MALDI-TOF MS spectrum of the peptide from Glu(Et)_2_ and nylon 4Me showed peaks with constant intervals of 157 g/mol derived from oligo(GluEt) with DP from 7 to 12 in Figure 2c. Peaks with an interval of 71 g/mol apart from the peaks derived from the oligo(GluEt) were also detected, thus confirming that the nylon 4 unit was introduced into the peptide. The ^1^H-NMR spectrum indicated that approximately 1 out of 10 oligo(GluEt) contained a nylon 4 unit (Figure 2d). The comparison between the reactions using nylon 4Et and nylon 4Me demonstrated that the differences in MS peak intervals were 85 g/mol and 71 g/mol apart from the peaks of oligo(GluEt) for nylon 4Et and nylon 4Me, respectively. If the nylon 4 unit were introduced at the N-terminus or the inner part of the resultant peptide, the molecular weight difference between the resultant products using nylon 4Et and nylon 4Me would be the same. Thus, we concluded that the nylon 4 unit was introduced at the C-terminus of oligo(GluEt). In the papain-catalyzed chemoenzymatic synthesis, the catalytic cysteine residue of papain forms a covalent bond with the carboxyl ester group of the monomer, thus resulting in formation of the enzyme-substrate (ES) complex (Scheme 1) [21]. The nucleophilic attack by the amine group of monomers allows peptide bond formation by aminolysis. It has been demonstrated that recognition of d-alanine and β-alanine by papain is approximately 100 times lower than that of l-alanine [16]. Although our reaction system contained nine times more nylon monomers than amino acids, introducing the nylon unit into the peptide was still difficult. Therefore, the nylon 4 unit was introduced only at the C-terminus. The results of this study and previous reports suggest that ES complex formation of the nylon 4 unit at the C-terminus with papain is not efficient enough to continue the polymerization (Scheme 1).

### 3.2. Bromelain, Proteinase K, and Lipase-Catalyzed Chemoenzymatic Synthesis Using Glu(Et)_2_ and Nylon 4Et

**Figure 3:**
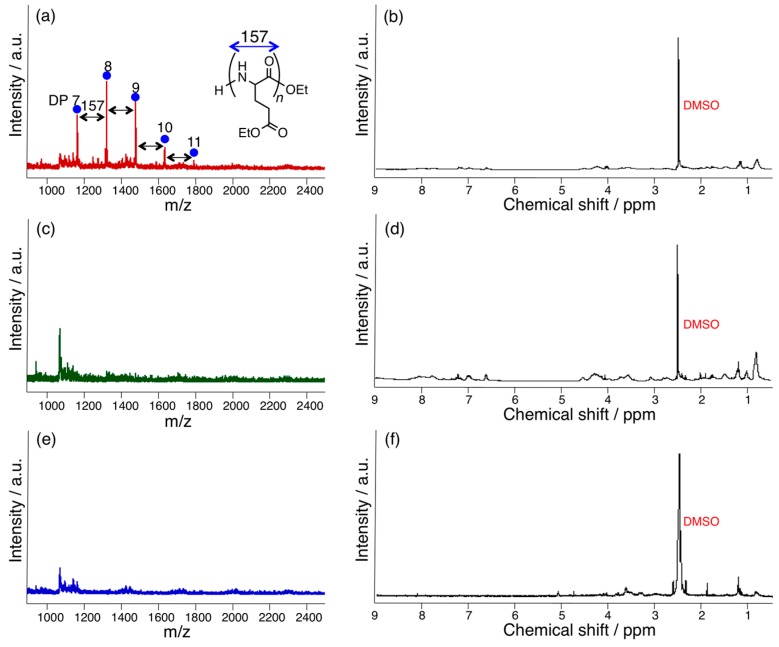
MALDI-TOF MS (**a**,**c**,**e**) and ^1^H-NMR (**b**,**d**,**f**) spectra of bromelain (**a**,**b**), proteinase K (**c**,**d**), and lipase (**e**,**f**)-catalyzed product using Glu(Et)_2_ and nylon 4Et as substrates. Mass spectra were obtained in linear positive mode using HABA and Na-TFA as the matrix and cationic reagent, respectively. Peaks with blue circles denote oligo(Glu) with various DP. DMSO-*d*_6_ was used as the solvent in NMR measurements.

To evaluate the possibility of using other enzymes to polymerize a nylon into peptides, we performed chemoenzymatic synthesis using the other enzymes, namely, bromelain, proteinase K, and lipase (CALB). We found that bromelain catalyzed the oligomerization of Glu(Et)_2_, but the nylon 4 unit was not introduced into the peptide according to MALDI-TOF MS and ^1^H-NMR spectra (Figure 3a,b). Bromelain is a cysteine protease like papain. Although the catalytic residues are identical in bromelain and papain, we reasoned that the difference in the binding sites for the substrates between bromelain and papain might affect the introduction of nylon 4 unit.

Proteinase K and lipase did not catalyze the oligomerization of glutamic acid or nylon introduction, according to MALDI-TOF MS (Figure 3c,e) and NMR spectra (Figure 3d,f). In the case of proteinase K, not only the catalytic triad Asp39-His69-Ser224 but also the stable substrate recognition site formed by Ca ions with Asp200, Val177, and Pro175 is required for the catalytic reactions [42]. In the case of lipase, the active center has a catalytic triad of Ser105-His224-Asp187 containing a large hydrophobic pocket above the catalytic triad and a medium-sized pocket below the catalytic triad [32]. It is assumed that the acyl moiety of the substrate lies in the large pocket, whereas the leaving group moiety lies in the medium pocket in the catalytic pathway [32]. In this study, Glu(Et)_2_ and nylon monomers were considered unable to meet the requirements for the catalytic pathways of proteinase K and lipase.

Papain has been reported to recognize β-alanine as a substrate in dipeptide synthesis [16]. It has been shown in a molecular modeling study that the extra carbon atom in β-alanine, compared with the natural amino acid, disrupts the hydrogen bonding network with Trp177 in papain, thus resulting in unstable recognition of β-alanine. The papain-catalyzed recognition rate of β-alanine has been estimated to be approximately 100 times lower than that of l-alanine [16]. In this study, we added nine-fold greater molar equivalents of nylon monomer than amino acid substrate in the chemoenzymatic reaction, and 13 mol % of the nylon unit was introduced into peptide by using papain. According to the resultant nylon composition and monomer feeding ratios, the papain-catalyzed recognition rate of the nylon 4 unit was also estimated to be approximately 100 times lower than that of Glu(Et)_2_.

### 3.3. Papain-Catalyzed Synthesis of Peptides with Nylon 1, 3, or 6 Monomers

**Figure 4:**
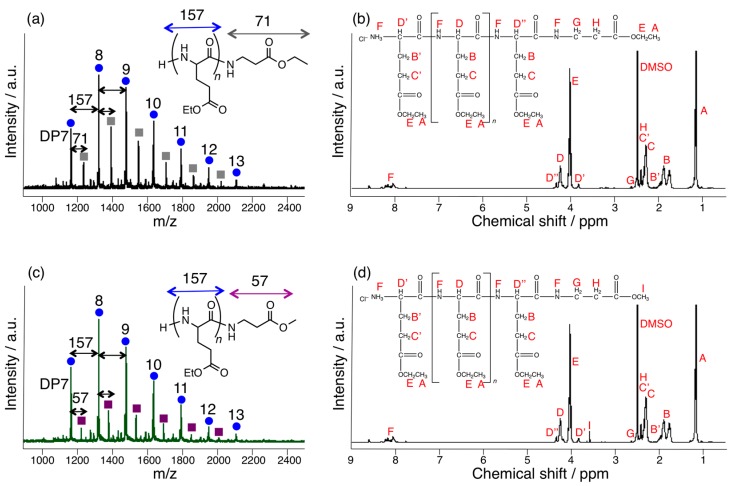
MALDI-TOF MS (**a**,**c**) and ^1^H-NMR (**c**,**d**) spectra of the papain-catalyzed reaction products using Glu(Et)_2_ with nylon 3Et (**a**,**b**) or nylon 3Me (**c**,**d**) as substrates. Mass spectra were obtained in linear positive mode using HABA and Na-TFA as the matrix and cationic reagent, respectively. Peaks with blue circles denote oligo(GluEt) with various DP, whereas peaks with gray (**a**) or purple (**c**) squares denote oligo(GluEt) with nylon 3Et or nylon 3Me, respectively. DMSO-*d*_6_ was used as the solvent in NMR measurements.

The papain-catalyzed substrate recognition using various nylon monomers, namely, nylon 1Et, nylon 3Me, nylon 3Et, and nylon 6Me, was studied by copolymerization with Glu(Et)_2_. In the cases of nylon 3Me and nylon 3Et, papain recognized and catalyzed nylon 3Et and nylon 3Me, and the nylon 3 unit was introduced into the C-terminus of the oligo(GluEt) according to the peaks with constant intervals of 71 and 57 g/mol apart from the peaks derived from the oligo(GluEt) in the MALDI-TOF MS spectra (Figure 4a,c). The yields were 49% ± 5% and 45% ± 2% when nylon 3Et and nylon 3Me were used as substrates, respectively. We did not find any peaks derived from peptides containing more than two nylon 3 units. The NMR spectra in Figure 4b,d indicated that the nylon compositions were 18 ± 4 mol % and 15 ± 6 mol % for nylon 3Et and nylon 3Me, respectively.

**Figure 5:**
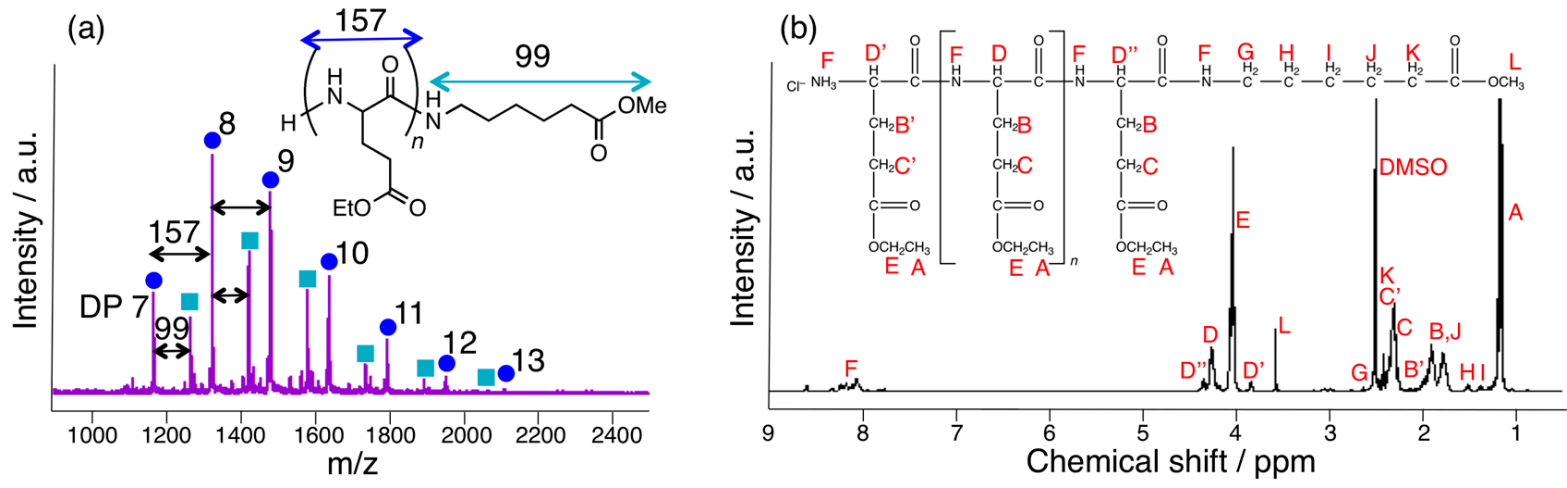
MALDI-TOF MS (**a**) and ^1^H-NMR (**b**) spectra of the papain-catalyzed reaction product using Glu(Et)_2_ with nylon 6Me as substrates. The mass spectrum was obtained in linear positive mode using HABA and Na-TFA as the matrix and cationic reagent, respectively. Peaks with blue circles denote oligo(GluEt) with various DP, and peaks with turquoise squares denote oligo(GluEt) with a nylon 6Me. DMSO-*d*_6_ was used as the solvent in NMR measurements.

Nylon 6Me was also investigated as a substrate of papain. Papain catalyzed the introduction of nylon 6Me with a yield of 35% ± 3%. Based on the MALDI-TOF MS spectrum shown in Figure 5a, nylon 6Me was introduced onto the C-terminus of oligo(GluEt). There were no peaks with more than two nylon 6 units. The NMR spectrum in Figure 5b shows that the monomeric composition of nylon 6 was 12 ± 2 mol % by using nylon 6Me and papain as the monomer and catalyst, respectively.

**Figure 6:**
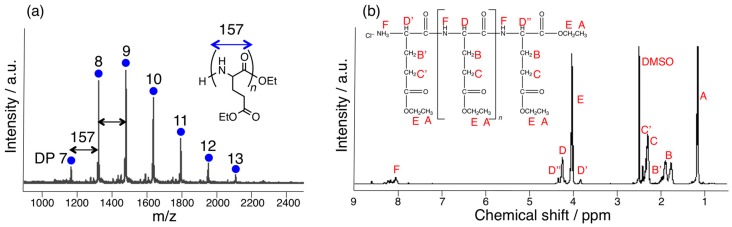
MALDI-TOF MS (**a**) and ^1^H-NMR (**b**) spectra of the papain-catalyzed reaction product using Glu(Et)_2_ and nylon 1Et as substrates. The MS spectrum was obtained in linear positive mode using HABA and Na-TFA as the matrix and cationic reagent, respectively. ^1^H-NMR was measured using DMSO-*d*_6_ as the solvent.

We performed papain-catalyzed synthesis using Glu(Et)_2_ and nylon 1Et. However, papain did not recognize nylon 1Et and synthesized oligo(GluEt) without nylon units in a yield of 40% ± 4%, according to the results by MALDI-TOF MS and ^1^H-NMR (Figure 6a,b). This finding was probably because nylon 1Et formed hydrogen bonds poorly with Asp158, Gly66, and Trp177, which is required for substrate affinity in papain [43]. In addition, ethyl carbamate that was used as nylon 1 in this study is estimated to have *pK*a of −1 [44] and less basic than primary amines. Therefore, the ethyl carbamate was probably difficult to react with catalytic cysteine residue of papain due to its low intrinsic nucleophilicity, thus being unable to form enzyme-substrate complex.

Based on the monomeric compositions, Table 1 lists the monomeric composition ratios of nylon units. The chain length and even-odd effects were not significant in the recognition efficiency of papain. The papain-catalyzed recognition rates of the nylon units were similar to that of nylon 4 and approximately 100 times lower than that of Glu(Et)_2_. To evaluate the recognition rate of nylon units in further detail, a more efficient copolymerization system for nylon and amino acids must be developed.

### 3.4. Thermal Analysis of oligo(GluEt) with or without Nylon Units

**Figure 7:**
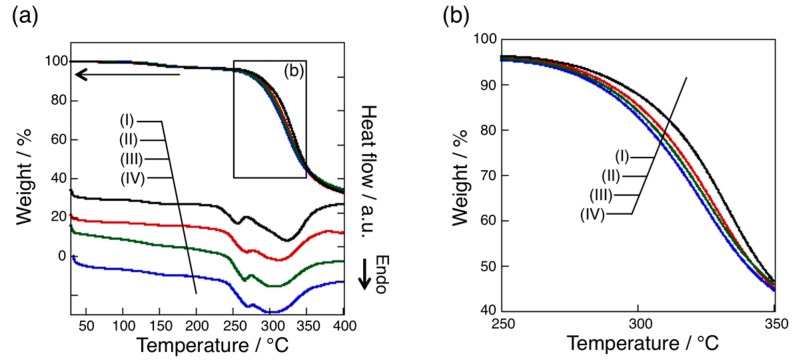
Thermal profiles of (I) oligo(GluEt); (II) oligo(GluEt-*co*-15 mol % nylon 3Me); (III) oligo(GluEt-*co*-12 mol % nylon 6Me); and (IV) oligo(GluEt-*co*-12 mol % nylon 4Me). (**a**) TGA and DSC from 30 to 400 °C at 20 °C/min; (**b**) Magnified TGA plot in the temperature range from 250 to 350 °C in (**a**).

To evaluate the effects of the nylon units on the thermal properties of oligo(GluEt), we performed TGA and DSC analyses of the resultant oligo(GluEt) with or without nylon units as shown in Figure 7a. On the basis of the DSC profiles, oligo(GluEt) with or without nylon units showed neither a transition temperature nor a melting temperature but demonstrated an endothermic degradation peak ranging from 230 to 350 °C (Figure 7a). The DSC profile of oligo(GluEt) slightly differed from those of the oligo(GluEt) containing nylon units: the degradation peaks of oligo(GluEt-*co*-15 mol % nylon 3Me), oligo(GluEt-*co*-12 mol % nylon 4Me) and oligo(GluEt-*co*-12 mol % nylon 6Me) shifted to higher temperatures in comparison with the profile of oligo(GluEt). On the basis of the weight loss in the TGA data (Figure 7b), the degradation temperatures were determined at 1, 5, and 10 wt % (Table 2). As a result, the oligo(GluEt) with nylon units demonstrated relatively lower thermal stability than oligo (GluEt) did. This result was probably because the intermolecular hydrogen bonds in the oligo(GluEt) with approximately 10 mol % of nylon units are prevented by the presence of the nylon units, and thus, the thermal stability of the oligo(GluEt) with nylon units decreased. 

## 4. Conclusions

In this study, we successfully introduced nylon 3, 4, and 6 units into the main chains of peptides through papain-catalyzed chemoenzymatic synthesis. The resultant copolymers demonstrated different thermal properties; thus, this study provides a first step toward controlling the thermal properties as well as adding a transition temperature of peptide-based polymers. The peptides containing nylon units are expected to have new functions, such as thermoplasticity, depending on controlling the density of the intermolecular hydrogen bonds. Thus, the papain-catalyzed introduction of nylon monomer into peptide reported in this study may be used more widely not only in peptide synthesis but also in material design in industrial application of peptides and poly(amino acid).

The nylon units were introduced at the C-termini in the resultant peptides because of the lower affinity of the nylon monomers with papain compared with natural amino acids such as glutamic acid. Generally, C-terminal modification is more challenging in comparison with protein N-terminal modification because side chains of aspartic and glutamic acid are as reactive as the general carboxylic acids in the amino acids. Therefore, this chemoenzymatic reaction could be a powerful tool in protein terminal modification, which is crucial in proteomics research.

## Figures and Tables

**Table 1:** Reaction yields and monomeric compositions of the nylon units in the papain-catalyzed synthesis with Glu(Et)_2_ (*n* = 3).

Nylon unit	Yield/mol %	Monomeric composition of nylon unit/mol %
Nylon 4Et	53 ± 2	13 ± 6
Nylon 4Me	45 ± 4	12 ± 3
Nylon 3Et	49 ± 5	18 ± 4
Nylon 3Me	45 ± 2	15 ± 6
Nylon 6Me	35 ± 3	12 ± 2
Nylon 1Et	40 ± 4	Not detected

**Table 2:** Degradation temperature at 1, 5, and 10 wt % weight loss of oligo(GluEt-*co*-15 mol % nylon 3Me), oligo(GluEt-*co*-12 mol % nylon 4Me), and oligo(GluEt-*co*-12 mol % nylon 6Me) (*n* = 3).

Product	1% Weight loss/°C	5% Weight loss/°C	10% Weight loss/°C
Oligo(GluEt-*co*-15 mol % nylon 3Me)	130 ± 6	265 ± 3	289 ± 1
Oligo(GluEt-*co*-12 mol % nylon 4Me)	145 ± 7	267 ± 9	289 ± 4
Oligo(GluEt-*co*-12 mol % nylon 6Me)	135 ± 7	267 ± 6	289 ± 4
Oligo(GluEt)	134 ± 6	271 ± 2	295 ± 1

## Abbreviations

The following abbreviations are used in this manuscript:

NMR	nuclear magnetic resonance
MALDI-TOF MS	matrix assisted laser desorption ionization time of flight mass spectrometry
nylon 4Me	methyl 4-aminobutyrate hydrochloride
nylon 4Et	ethyl 4-aminobutyrate hydrochloride
nylon 3Me	β-alanine methyl ester hydrochloride
nylon 3Et	β-alanine ethyl ester hydrochloride
nylon 6Me	methyl 6-aminohexanoate hydrochloride
nylon 1Et	ethyl carbamate
glu(Et)_2_	l-glutamic acid diethyl ester hydrochloride
oligo(GluEt)	oligo(glutamic acid-γ-ethyl ester)
DP	degree of polymerization
HABA	2-(4-hydroxyphenylazo)benzoic acid
Na-TFA	sodium trifluoroacetate
DMSO-*d*6	dimethyl sulfoxide-*d*_6_
